# A case report of recurrent testicular germ cell tumor in a patient with a history of primary pulmonary germ cell tumor and a review of the literature

**DOI:** 10.3389/fonc.2024.1361380

**Published:** 2024-07-09

**Authors:** Jian Tan, Jinfeng Wu, Runqiang Yuan, Wei Li, Linfeng Li, Hongxing Huang, Yangbai Lu

**Affiliations:** ^1^ Department of Urology, Zhongshan People's Hospital, Zhongshan, China; ^2^ First Clinical Medical College, Guangdong Medical University, Zhanjiang, China

**Keywords:** germ cell tumors, extra-gonadal germ cell tumors, primary pulmonary germ cell tumor, non-seminoma germ cell tumor, case report

## Abstract

**Background:**

Compared to testicular germ cell tumors, the incidence of extragonadal germ cell tumors (EGCTs) is relatively low. While the lungs are a common site for metastasis of malignant germ cell tumors, primary pulmonary germ cell tumors are extremely rare.

**Objective:**

To enhance the understanding of the diagnosis and treatment of germ cell tumors, particularly extragonadal germ cell tumors (EGCTs).

**Methods:**

A Case Report of Recurrent Testicular Germ Cell Tumor in a Patient with Primary Pulmonary Germ Cell Tumor and a Review of the Literature.

**Clinical data:**

The patient was initially diagnosed with primary pulmonary germ cell tumor and received standard treatment. Five years later, the patient developed a recurrent testicular germ cell tumor. The pathological results from the two surgeries were different, indicating embryonal carcinoma in the first instance and seminoma in the second.

**Conclusion:**

For cases with a high suspicion of extragonadal germ cell tumors (EGCTs), early pathological biopsy is essential to confirm the histological subtype and to guide the selection of the most appropriate and sensitive treatment regimen.

## Introduction

Germ cell tumors (GCTs) are neoplasms that arise from germ cells, occurring either in the gonads or extragonadal sites. Extra-gonadal germ cell tumors (EGCTs) are malignant germ cell tumors that occur outside the testes and ovaries, typically in midline anatomical locations. EGCTs constitute only 3–5% of all germ cell tumors and predominantly affect adolescent males. The specific anatomical sites of occurrence vary with age. In adults, the most common sites are, in order of frequency: the anterior mediastinum, the retroperitoneum, and the pineal or suprasellar regions (1, 2). The incidence of extragonadal germ cell tumors (EGCTs) is relatively low, and occurrences in the lungs are even rarer. Currently, there are few related reports in both domestic and international literature. Therefore, this paper presents a case of primary pulmonary germ cell tumor with subsequent recurrence as testicular germ cell tumor, treated at Zhongshan People’s Hospital (hereinafter referred to as our hospital). The aim is to enhance understanding and recognition of the diagnosis and treatment of germ cell tumors, particularly extragonadal germ cell tumors.

## Case presentation

### Patient information: A 25-year-old male

Clinical History: The patient was admitted in September 2018 due to the discovery of a shadow in the lower right lung that had been present for over a month. The patient did not report any symptoms such as coughing, sputum production, or hemoptysis.

Physical Examination: The patient’s height was 165 cm, weight was 66 kg, and BMI was 24.24 kg/m². A comprehensive physical examination revealed no significant abnormalities. The patient denied any family history of genetic disorders, history of cryptorchidism, or testicular trauma.

### Diagnostic assessment

**Figure 1:**
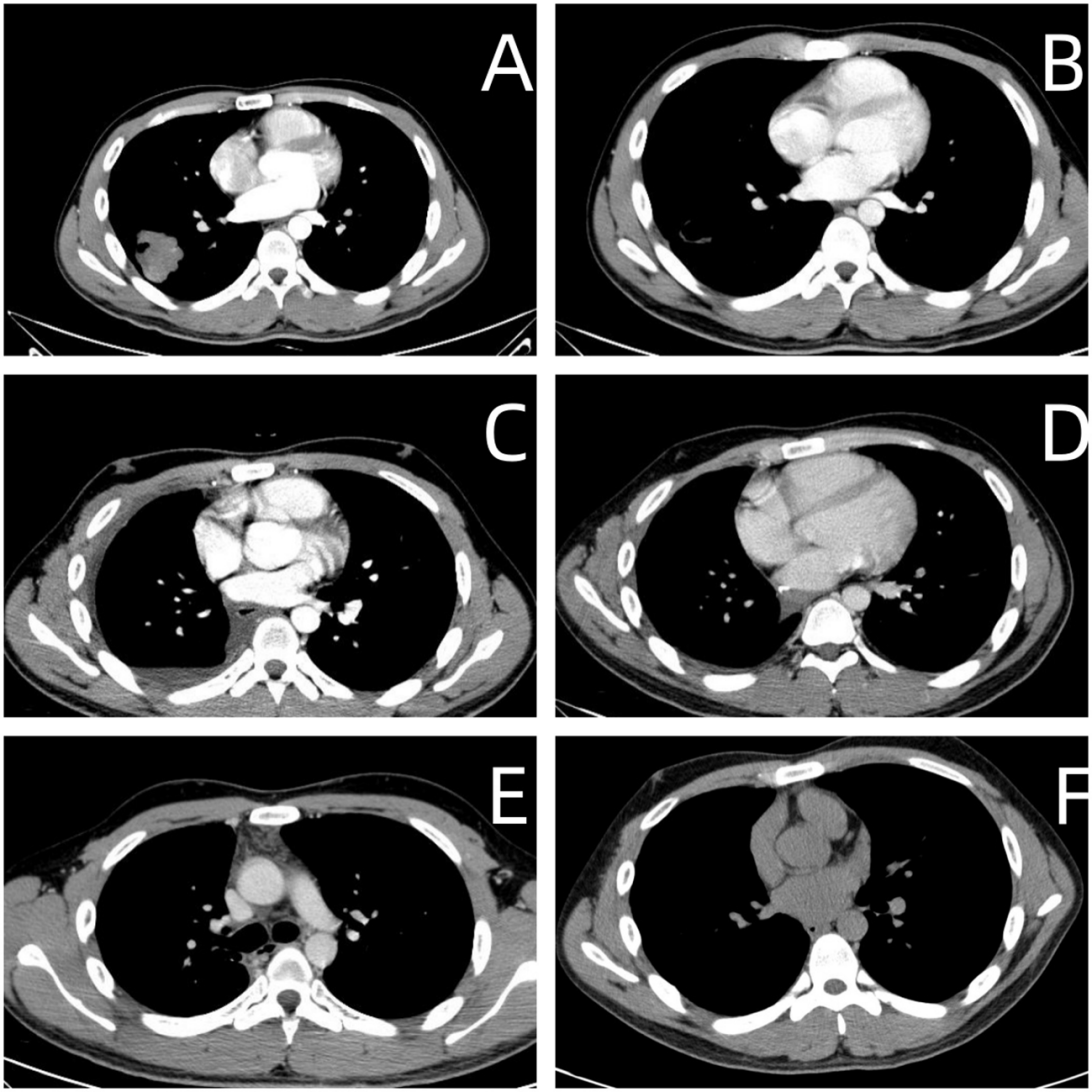
**(A)** Upon Hospital Admission. • Imaging revealed a lobulated shadow in the right lower lung field, measuring approximately 3.6 cm × 4.4 cm, with cavitation observed within. **(B)** After Preoperative Chemotherapy (Before Right Lung Lobectomy). • Imaging showed a reduction in the right lower lung mass to 2.0 cm × 1.8 cm, now presenting as a cavitary lesion. **(C)** One Month Post-Right Lung Lobectomy. • Imaging indicated postoperative changes in the right lung without any abnormal density. **(D)** Three Months Post-Right Lung Lobectomy. • Imaging showed postoperative changes in the right lung with no abnormal density observed. **(E)** Six Months Post-Right Lung Lobectomy. • Imaging demonstrated postoperative changes in the right lung with no abnormal density noted. **(F)** Five Years Post-Right Lung Lobectomy. • Imaging revealed postoperative changes in the right lung, with no abnormal density detected.

**Figure 2:**
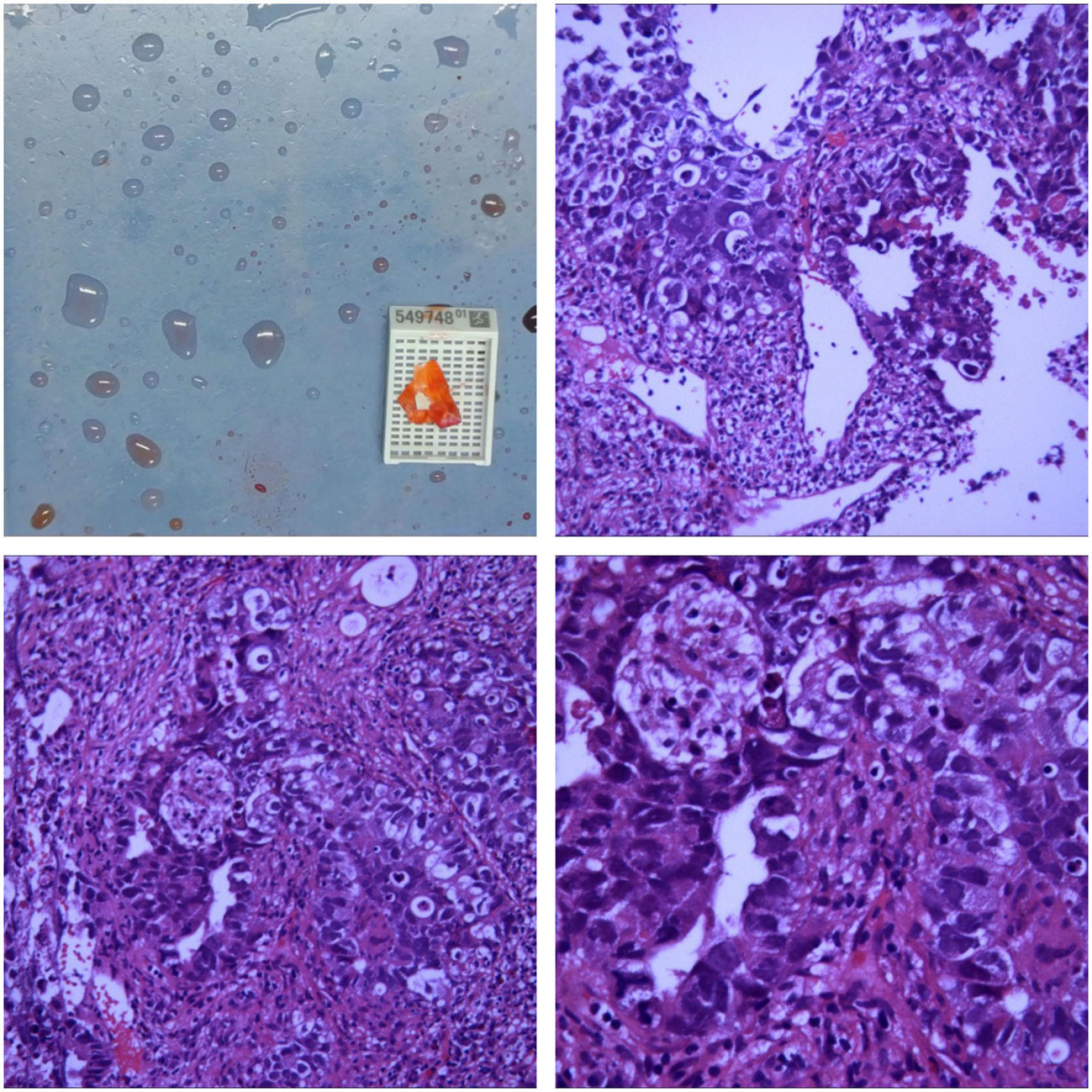
Microscopic Findings The biopsy specimen reveals atypical tumor cells arranged in nests, with some areas showing glandular-like infiltrative growth. The cells exhibit significant pleomorphism, with large, hyperchromatic nuclei. There is a notable presence of inflammatory cell infiltration and scattered multifocal necrosis. Immunohistochemistry Results CK +, CK5/6 -, CK7 -, P63 -, CK20 -, TTF-1 -, GATA3 -, CD30 +, S-100 -, SALL4 +, CD56 -, chromogranin A -, Syn -. Conclusion Initial assessment of the right lung tissue suggests a high likelihood of malignancy, with a strong suspicion of non-small cell carcinoma (NSCLC).Based on the immunohistochemistry results, the poorly differentiated malignant tumor in the right lower lung is highly suggestive of a germ cell tumor.

**Figure 3:**
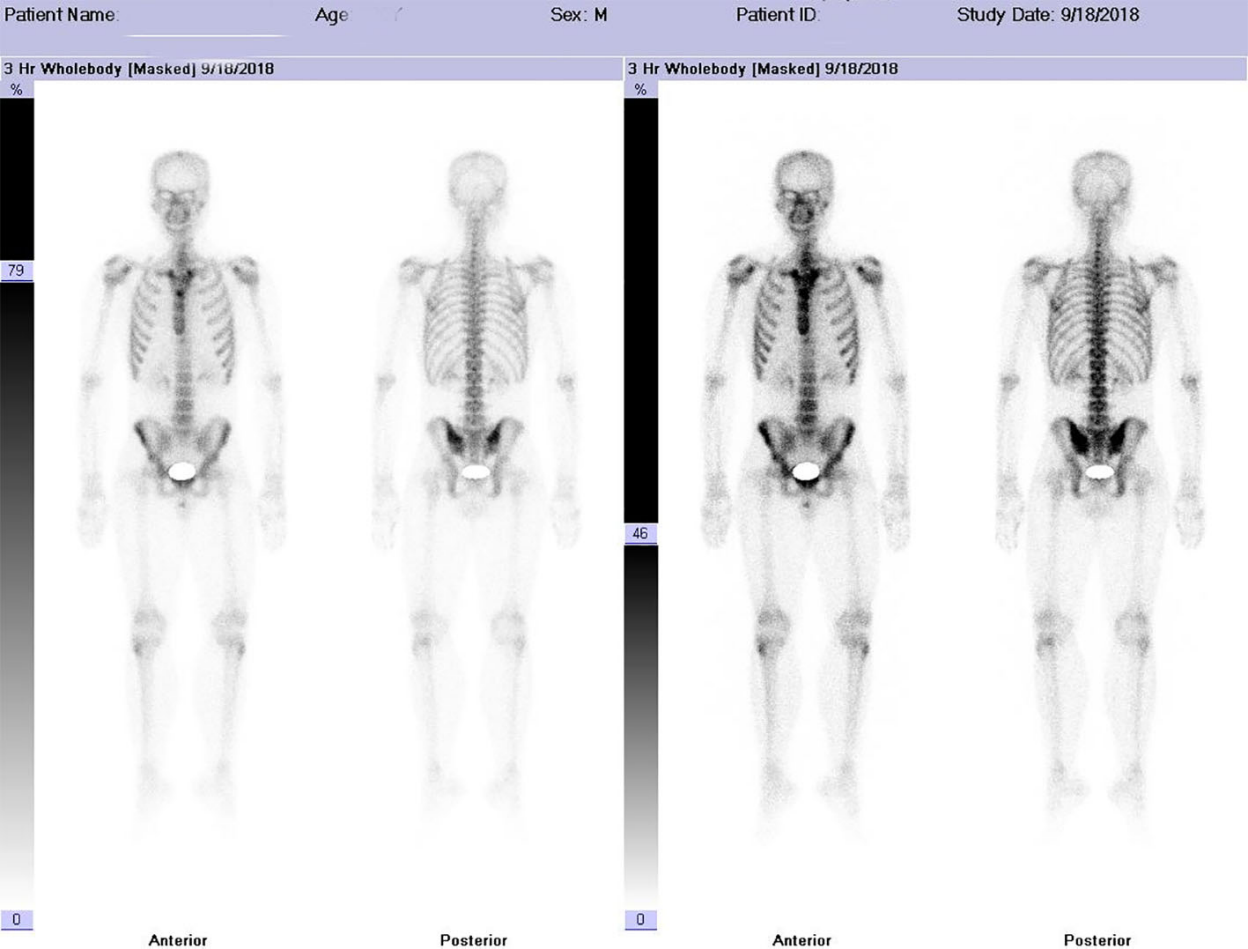
Imaging Findings Intravenous injection of 99m-Tc-MDP was administered, and planar whole-body skeletal imaging was performed in both anterior and posterior views two hours post-injection. The results are as follows: • The whole-body bone scan is clear, with normal morphology and structure. • The radiotracer distribution in the bones is generally uniform and symmetrical, with no localized areas of abnormal radiotracer uptake or defects observed. • Both kidneys are visible in the images. Imaging Conclusion The whole-body skeletal metabolic imaging appears normal, with no evidence of bone metastasis from malignant tumors.

**Figure 4:**
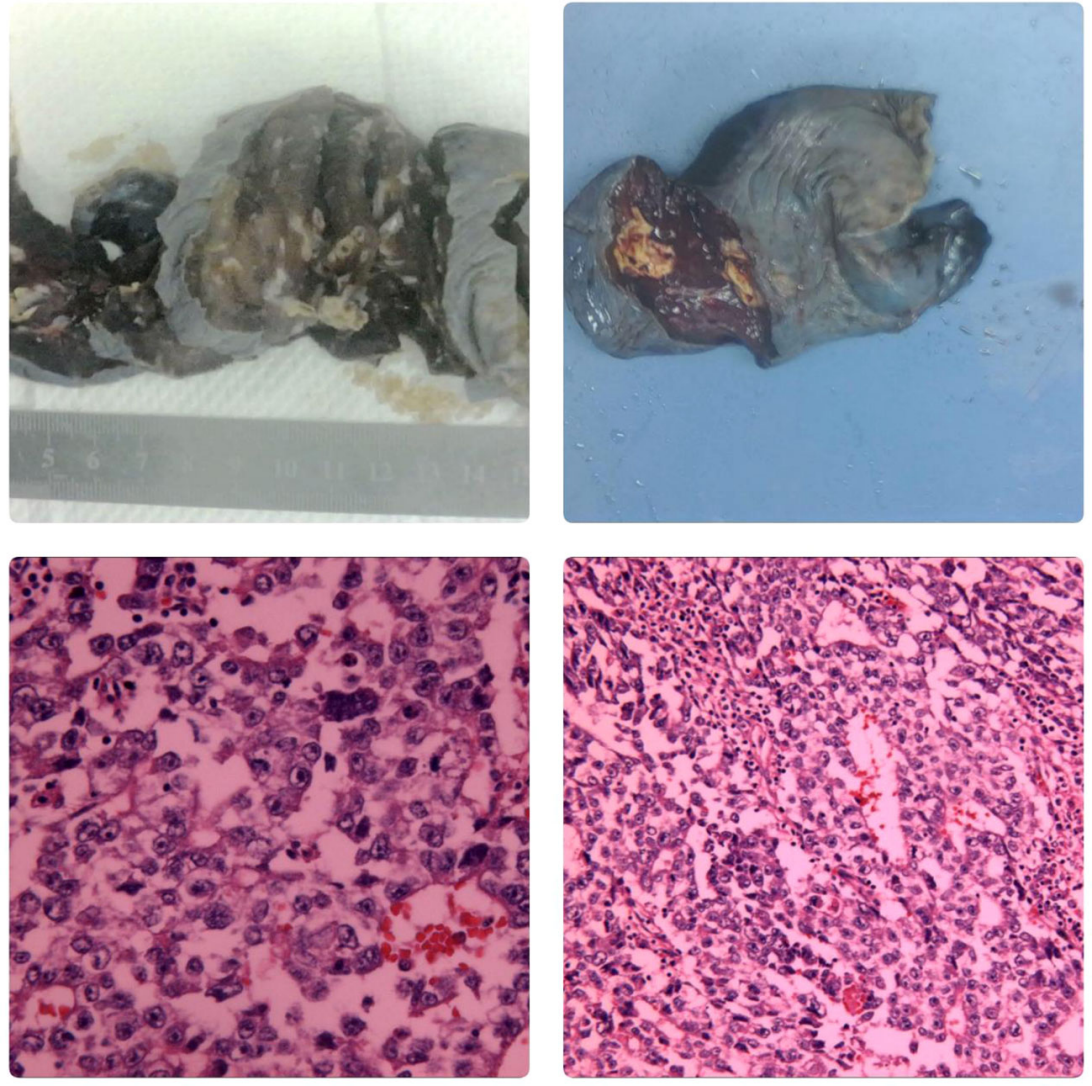
Macroscopic Examination The right lower lung lobe specimen measured 12x8x6 cm. A nodule, approximately 2 cm in diameter, was observed 3.5 cm from the resection margin and 0.3 cm beneath the pleural surface. The nodule was grayish-yellow, well-defined, and soft, with a cystic appearance on the cut surface. Microscopic Examination Hematoxylin and eosin (HE) staining revealed nests of atypical tumor cells, some resembling glandular structures infiltrating the surrounding tissue. The tumor cells varied in size, with large, hyperchromatic nuclei. There was significant infiltration of inflammatory cells surrounding the tumor, and multifocal necrosis was observed. Immunohistochemistry CK+, SALL4+, PLAP+, CK19+, CD30+, AFP+/-, Hepatocyte-OCT3/4-, HCG-, CDX2-, CEA-, CD5-, CD117-, TdT-. *In situ* hybridization EBER-. Conclusion: embryonal carcinoma In October 2023, the patient was admitted to our hospital due to “right testicular enlargement for more than half a month.”On physical examination, the patient was 171 cm tall and weighed 69.6 kg, with a BMI of 23.80 kg/m². The right testicle was enlarged to approximately the size of a goose egg, with a firm texture, no obvious tenderness, and no signs of local redness, heat, or pain. A multislice enhanced CT scan of the testes/epididymis at our hospital revealed an enlarged right testicle with a solid mass, suggesting a high possibility of a malignant tumor (**Figure 5**). Serum levels of AFP, β-HCG, and LDH were all within normal ranges. A multi-slice enhanced CT scan of the chest showed no signs of tumor recurrence or metastasis. On October 12 of the same year, the patient underwent a right radical orchiectomy. Intraoperatively, the testis measured 7x6.8x6 cm, and a tumor measuring 6x5x4 cm was observed upon sectioning. Postoperative pathological staging was T1bN0M0, with a clinical stage of I. Pathology indicated a diagnosis of seminoma in the right testis (**Figure 6**). In 2024, the patient received antitumor treatment with carboplatin 500mg on December 12, 2023, and January 3, 2024. A follow-up examination in March 2024, six months post-surgery, included a whole-body spiral CT scan with contrast enhancement of the lungs and testes, as well as tests for the three aforementioned serum tumor markers. All results were normal (**Figure 1**, **Table 1**). We believe that clinical cure has been achieved.

A contrast-enhanced spiral CT scan of the chest performed at our hospital indicated a malignant tumor in the lower right lung, with metastasis to the mediastinum and lymph nodes (**Figure 1**). In the same month, a biopsy of the right lower lung mass was performed, and the pathological results indicated a germ cell tumor (**Figure 2**). A detailed re-examination of the external genital system revealed no testicular enlargement or tenderness. A complete urinary system ultrasound showed that the size and morphology of both seminal vesicles were normal, with homogeneous internal echoes and no significant abnormal echoes. Serum AFP and β-HCG levels were approximately normal, while LDH was mildly elevated (< 1.5 times the normal value) (**Table 1**). A contrast-enhanced CT scan of the entire abdomen and pelvis showed no abnormalities. After excluding the possibility of non-testicular primary or metastatic germ cell tumors, our hospital diagnosed the patient with “primary pulmonary germ cell tumor.” The preoperative evaluation, including a comprehensive ECT (electrocardiogram and cardiac test), revealed no significant abnormalities (**Figure 3**) count showed no significant abnormalities. After excluding contraindications for chemotherapy, the patient began BEP regimen chemotherapy on September 30th of the same year (Bleomycin 30,000U on Days 1, 8, and 15; Etoposide 170mg on Days 1–5; Cisplatin 30mg on Days 1–5). Post-treatment follow-up with contrast-enhanced spiral chest CT indicated a significant reduction in the original right lower lung mass, now confined to the right lower lobe (**Figure 1**). After excluding contraindications for surgery, the patient underwent a thoracoscopic right lower lobectomy with hilar and mediastinal lymph node dissection on December 4th. Intraoperative examination of the resected specimen revealed a hard, yellowish-white mass. Postoperative pathology indicated a germ cell tumor, with a strong suggestion of embryonal carcinoma (**Figure 4**). Following surgery, the patient underwent four courses of postoperative BEP chemotherapy on December 29th of the same year, and on January 19th, February 12th, and March 6th of the following year, with the chemotherapy regimen remaining the same as previously administered. A follow-up examination six months post-surgery (June 2019) included a full spiral enhanced CT scan of the lungs and tests for the three aforementioned serum tumor markers, all of which returned normal results (**Figure 1**, **Table 1**). We believe that clinical cure has been achieved.

**Table 1:** During both occurrences of the disease, AFP and β-HCG levels remained within the normal reference range.

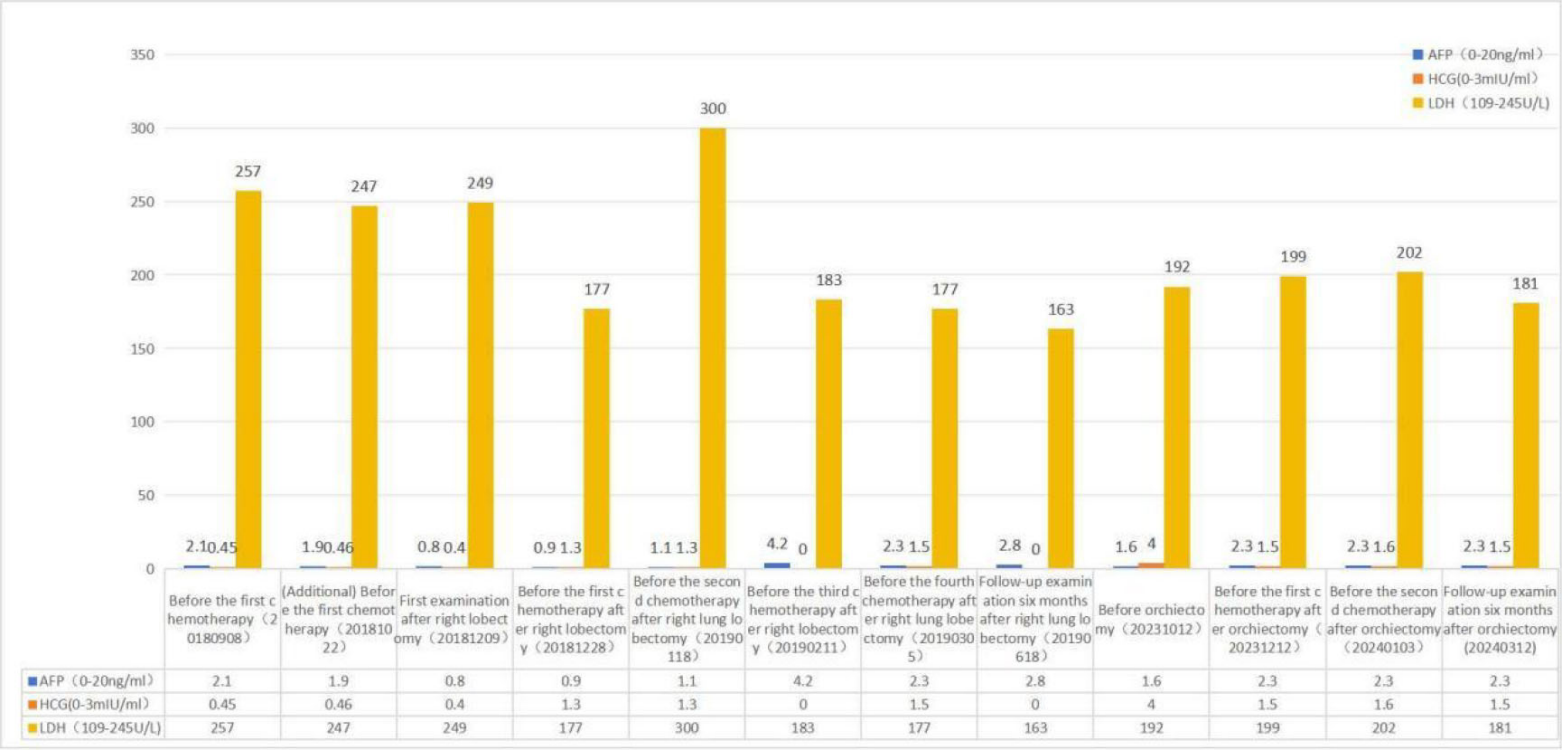

LDH levels were slightly elevated above the normal range (less than 1.5 times the upper limit of normal) only during the first occurrence.

**Figure 5:**
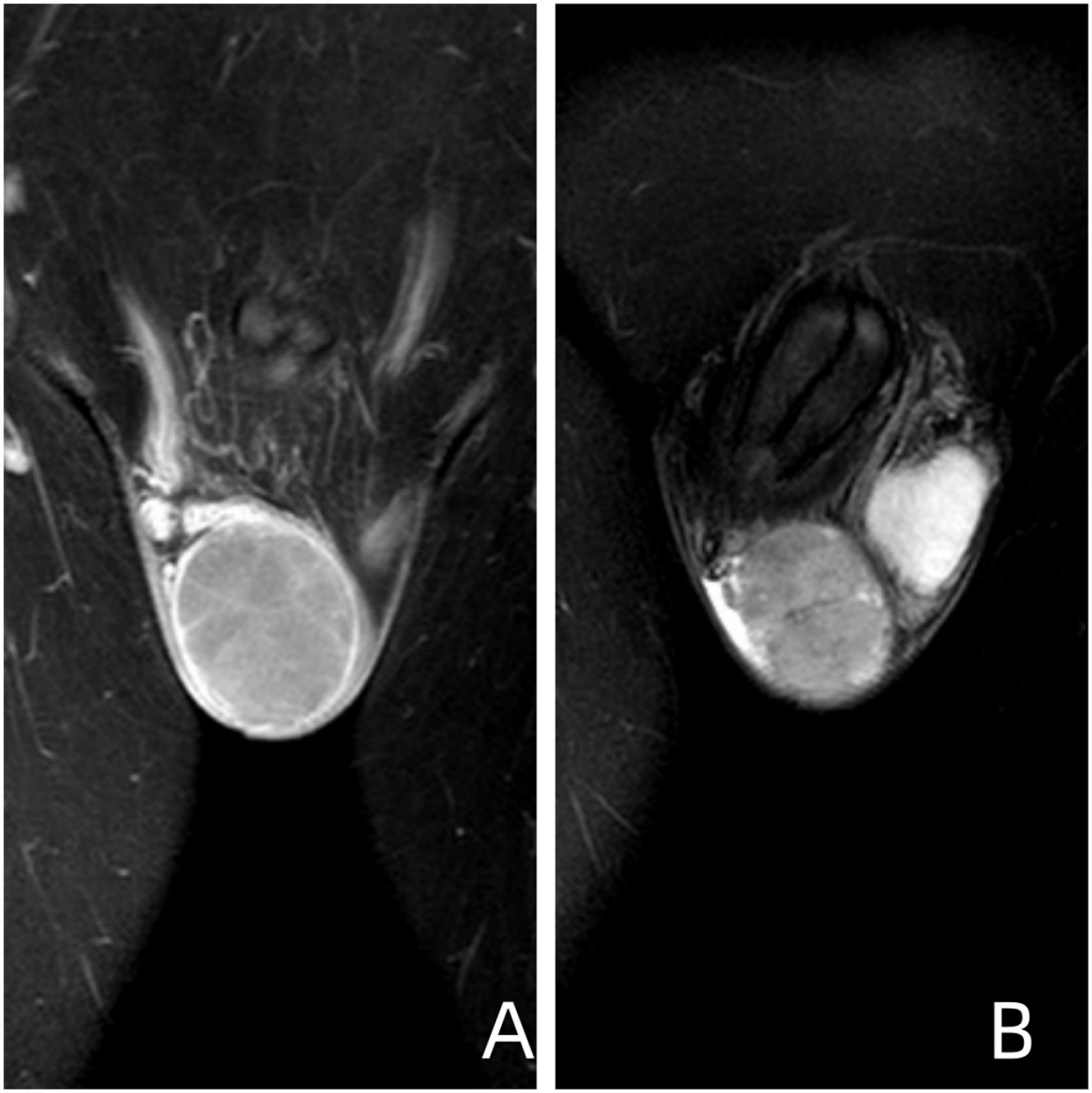
Image Descriptions and Findings **(A)** Coronal T1-weighted image of the testis obtained using contrast-enhanced multi-slice spiral CT. **(B)** Coronal T2-weighted fat-suppressed image of the testis. Imaging Conclusion: The right testis exhibits a roughly spherical mass measuring approximately 47mm x 52mm x 53mm. The findings are suggestive of embryonal carcinoma.

**Figure 6:**
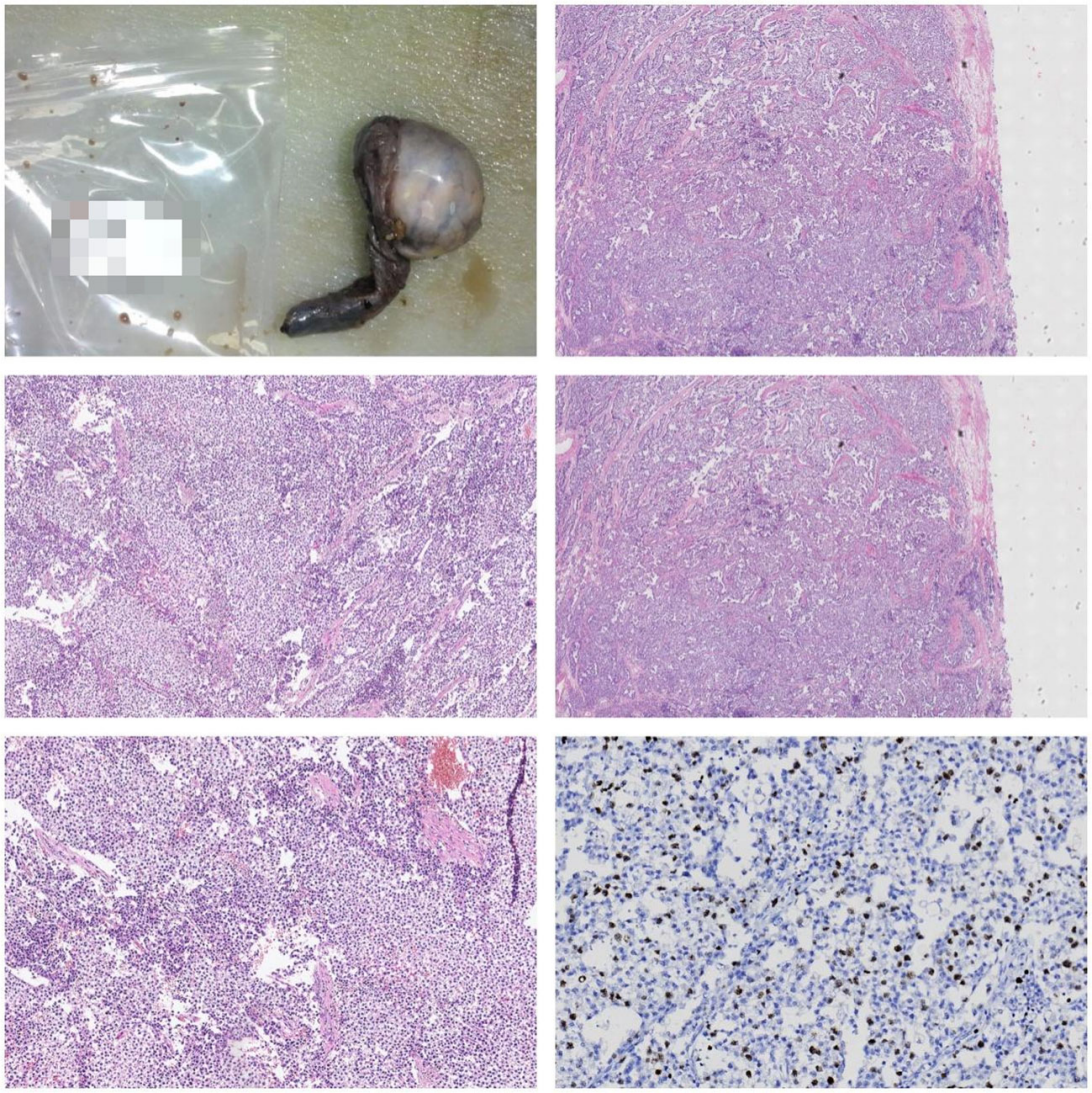
Gross Description: The right testis and tumor: The testis measures 7 x 6.8 x 6 cm. Upon sectioning, a 6 x 5 x 4 cm tumor is observed with a graywhite, solid appearance. The tumor has well-defined borders and is adjacent to the capsule. The epididymal structure is not clearly defined. The spermatic cord is 7 cm long, with a dark red cut surface. Microscopic Description: Under the microscope, the tumor is composed of uniformly shaped primitive cells arranged in diffuse sheets. These sheets are separated by delicate fibrous septa, forming lobular and nested patterns. Focal areas of necrosis are present. Immunohistochemistry: S-100 (-) Ki-67 (10%) HCG (-) CD30 (-) CD117 (+) PLAP (-) CK (-) AFP (-) Pathological Diagnosis: Seminoma of the right testis.

## Discussion

### Epidemiology

The incidence of extragonadal germ cell tumors (EGCTs) is relatively low. According to the 2022 WHO classification of urinary system tumors, EGCTs account for only 3–5% of all germ cell tumors. As reported by Karami et al. (3, 4), Extragonadal germ cell tumors (EGCTs) occur in 54% of cases in the mediastinum and 45% in the retroperitoneum, with other locations accounting for only 1%. Reports of primary pulmonary germ cell tumors are exceedingly rare. Following seminoma, embryonal cell carcinoma (EC) is the second most common type of germ cell tumor. EC primarily affects individuals between the ages of 20 and 30 (5, 6).

### Pathogenesis

The pathogenesis of extragonadal germ cell tumors (EGCTs) remains unclear. However, there are currently two main hypotheses. The first hypothesis suggests that (7) during embryonic development, primordial germ cells (PGCs) may deviate from their normal migratory path and evade immune system clearance. Typically, PGCs migrate from the yolk sac wall to the genital ridge along the path of sympathetic nerve fibers, eventually forming the gonads. However, if PGCs enter the neural branches at the gonadal site, they may continue to migrate along the midline of the body via the sympathetic nerve trunk, potentially reaching and residing in distant locations such as the retroperitoneum, abdomen, anterior mediastinum, neck, and midline of the brain. By escaping apoptosis, these mislocated PGCs can survive and progress to extragonadal germ cell tumors (EGCTs) in these various locations (8, 9). Transformed germ cells in the testes, due to various traumas such as burns and blunt injuries, may undergo retrograde migration from their primary site and eventually progress to extragonadal germ cell tumors (EGCTs). It has also been reported that 80% of testicular germ cell tumors exhibit characteristic chromosomal abnormalities, including sex chromosome anomalies such as Klinefelter syndrome (47, XXY) and autosomal abnormalities such as isochromosome 12p (i12p) (10, 11). Mutations in the CCND2 and RB1 genes have been associated with the development of extragonadal germ cell tumors (EGCTs) found in the mediastinum. Additionally, mutations in the PRDM14 gene have also been identified in EGCTs and are believed to be related to the origin and progression of these tumors (8, 12). Due to the sequential occurrence of germ cell tumors in the lungs and testes in this case, it was highly suspected that chromosomal abnormalities might be the underlying cause. However, the patient declined chromosomal testing for personal reasons, and ultimately, such tests were not performed.

### Diagnosis

For germ cell tumors (GCTs), the symptoms are typically more distinct and apparent compared to extragonadal germ cell tumors (EGCTs). Patients often present with a solitary, painless, hard mass in the affected side of the scrotum, sometimes accompanied by a sensation of scrotal heaviness, leading to timely diagnosis and treatment. In contrast, early-stage EGCTs are challenging to diagnose promptly due to their lack of specific symptoms, imaging features, and tumor markers. Most EGCTs are diagnosed at an advanced stage when patients present with symptoms related to mass effects in the mediastinum, brain, or other locations. Li Ping et al. (13) highlighted that the misdiagnosis of extragonadal germ cell tumors (EGCTs) is relatively common. The 2022 edition of the Chinese Guidelines for the Diagnosis and Treatment of Urological and Andrological Diseases emphasizes the significant value of serum tumor markers, including alpha-fetoprotein (AFP), beta-human chorionic gonadotropin (β-HCG), and lactate dehydrogenase (LDH), in the diagnosis and prognosis of testicular tumors. However, according to Murray et al. (14), For 70% of embryonal carcinoma cases, serum AFP levels are elevated. In contrast, serum AFP levels typically remain normal in seminoma cases. Elevated serum β-HCG levels are often observed when the tumor contains syncytiotrophoblastic cells. In seminoma patients, only 15%-20% of those in the advanced stages exhibit elevated serum β-HCG levels. For early-stage non-seminomatous germ cell tumor (NSGCT) patients, only 10%-20% show elevated serum β-HCG levels, whereas in advanced-stage NSGCT patients, the percentage increases to 40%. Therefore, for patients presenting with insidious onset of lung involvement and markedly elevated serum tumor markers, a high suspicion of germ cell tumors necessitates timely pathological biopsy. Additionally, ECT or PET-CT is recommended to assess lymph node metastasis and distant invasion. However, in this particular case, throughout two disease progressions and the entire treatment process, only mild elevation of LDH was observed, and notably, β-HCG levels remained normal. This had an impact on the initial diagnosis: on one hand, germ cell tumors were not considered in the differential diagnosis, and on the other hand, it highlighted the limitations in sensitivity and specificity of the three serum markers, thus hindering their broader application. According to Murray (15), new biomarkers such as the levels of circulating miR-30b-5p in peripheral blood hold promising potential for the diagnosis and prognosis of such diseases.

### Treatment

Reports on primary pulmonary germ cell tumors are rare, and there is a lack of treatment experience. This case involves a pulmonary mass identified as embryonal carcinoma, a clinically rare and highly malignant type of germ cell tumor. Embryonal carcinoma is prone to early metastasis and recurrence, with nearly half of the patients presenting at an advanced stage at the time of diagnosis (1, 16). Embryonal carcinoma is generally limited in its responsiveness to radiotherapy and is typically not the primary mode of treatment. Instead, radiotherapy is often used as an adjunct to chemotherapy to control tumor progression. Embryonal carcinoma cells exhibit high sensitivity to chemotherapy due to their rapid rate of division. Chemotherapy is highly effective in killing these rapidly dividing cells, often leading to a significant reduction in tumor burden. In many cases, chemotherapy can even achieve complete remission. Notably, preoperative radiotherapy can result in substantial tumor shrinkage, consistent with findings from previous studies (17, 18). Based on factors such as histological type, staging, and performance status, we opted for a treatment regimen consisting of preoperative and postoperative chemotherapy combined with surgical resection. The chemotherapy regimen was platinum-based, and the surgical procedure involved lobectomy rather than wedge resection. According to Ginsberg et al. (19, 20), The local recurrence rate following wedge resection is approximately three times higher than that of lobectomy (5.4% vs. 1.9%), with a trend towards lower survival rates. During a five-year follow-up after discharge, no tumor recurrence, metastasis, or other adverse outcomes were observed, demonstrating the efficacy of this treatment approach. It is noteworthy that during the review of the patient’s treatment courses, the patient experienced significant chemotherapy-related adverse effects, particularly vomiting, as reported by Shah et al. (21), Carboplatin has lower toxicity compared to cisplatin, with a comparable five-year disease-free survival rate. Patients treated with carboplatin experience less severe and less frequent vomiting than those treated with cisplatin. In first-line therapy, it is important to consider whether adjusting the chemotherapy regimen could reduce patient discomfort during treatment. According to a report by Massard, C., et al. (22), For patients whose serum AFP, β-HCG, and LDH levels fluctuate around critical values, those with levels consistently below these thresholds tend to experience slower disease progression and generally have a better prognosis.

## Conclusion

This case has the following features: a young male patient who developed primary pulmonary germ cell tumor and testicular germ cell tumor within a span of five years. Unlike the diagnosis and treatment of testicular germ cell tumors, the pulmonary symptoms and signs in this case were atypical. The serum markers recommended by the guidelines were also negative. Considering the rarity of primary pulmonary germ cell tumors in previous reports, the pathological types are complex, and there is limited understanding of their radiological features with no specific imaging characteristics. Preoperative pathological biopsy remains the gold standard for diagnosing such diseases. Currently, there is no definitive treatment protocol for early-stage primary pulmonary germ cell tumors. However, based on the pathological type, we initially chose the BEP regimen (Bleomycin, Etoposide, and Cisplatin) for chemotherapy. After significant tumor reduction, radical surgical resection of the lesion was performed, followed by four additional cycles of adjuvant chemotherapy with the same regimen. Even in previous reports, the disease-free survival (DFS) rates for extragonadal germ cell tumors (EGCTs) are typically as follows: For stage I, the five-year DFS is usually over 90%; for stage II, with surgery and adjuvant chemotherapy, the five-year DFS ranges from 70–85%; for stage III, the prognosis is relatively poor, with a five-year DFS between 50–70% (23, 24). Unlike typical germ cell tumors, extragonadal germ cell tumors do not respond as well to radiotherapy and chemotherapy. However, in this case report, we followed the patient for five years and observed no recurrence or metastasis. This suggests that considering histological type and staging to select treatment regimens similar to those used for sensitive germ cell tumors can often result in favorable outcomes when applied early.

## Data availability statement

The original contributions presented in the study are included in the article/supplementary material. Further inquiries can be directed to the corresponding author.

## Ethics statement

The study was approved by the Institutional Review Board and the Ethics Committee of the Zhongshan City People’s Hospital and written informed consent was obtained from this patient. Written informed consent was obtained from the participant/patient(s) for the publication of this case report.

## Author contributions

JT: Funding acquisition, Writing – review & editing, Writing – original draft. JW: Data curation, Investigation, Writing – review & editing. YL: Conceptualization, Writing – review & editing, Supervision. WL: Writing – review & editing, Investigation, Conceptualization. LL: Writing – review & editing. RY: Methodology, Data curation, Writing – review & editing. HH: Writing – review & editing, Visualization, Validation, Supervision.
